# Clinical profile, treatment and outcome of pediatric brain tumors in Serbia in a 10-year period: A national referral institution experience

**DOI:** 10.1371/journal.pone.0259095

**Published:** 2021-10-26

**Authors:** Dragana Stanić, Danica Grujičić, Tatjana Pekmezović, Jelena Bokun, Marija Popović-Vuković, Dragana Janić, Lejla Paripović, Vesna Ilić, Marija Pudrlja Slović, Rosanda Ilić, Savo Raičević, Milan Sarić, Ivana Mišković, Borko Nidžović, Marina Nikitović

**Affiliations:** 1 Faculty of Medicine, University of Belgrade, Belgrade, Serbia; 2 Pediatric Radiation Oncology Department, Institute of Oncology and Radiology of Serbia, Belgrade, Serbia; 3 Neuro-Oncology Department, Clinic of Neurosurgery, Clinical Center of Serbia, Belgrade, Serbia; 4 Pediatric Oncology Department, Institute of Oncology and Radiology of Serbia, Belgrade, Serbia; 5 Institute of Epidemiology, Faculty of Medicine, University of Belgrade, Belgrade, Serbia; 6 Medical Physics Department, Institute of Oncology and Radiology of Serbia, Belgrade, Serbia; CHOC Children’s Hospital - UC Irvine, UNITED STATES

## Abstract

**Objective:**

This study aimed to evaluate the characteristics of children with primary brain tumors, the effectiveness of treatment modalities, and to detect factors related to the outcome.

**Methods:**

A detailed analysis was performed on a series of 173 pediatric patients treated in a Serbian referral oncology institution between 2007 and 2016, based on their clinical, histological, treatment, and follow-up data.

**Results:**

Mean survival time of all children was 94.5months. 2-, 5- and 10-year overall survival probabilities were 68.8%, 59.4%, and 52.8%, respectively. Patients with supratentorial tumors had longer survival than patients with infratentorial tumors and patients with tumors in both compartments (p = 0.011). Children with the unknown histopathology (brainstem glioma) and high-grade glioma had a shorter life than embryonal tumors, ependymoma, and low-grade glioma (p<0.001). Survival of the children who underwent gross total resection was longer than the children in whom lesser degrees of resection were achieved (p = 0.015). The extent of the disease is a very important parameter found to be associated with survival. Patients with no evidence of disease after surgery had a mean survival of 123 months, compared with 82 months in patients with local residual disease and 55 months in patients with disseminated disease (p<0.001). By the univariate analysis, factors predicting poor outcome in our series were the presentation of disease with hormonal abnormalities, tumor location, and the extent of the disease, while the factors predicting a better outcome were age at the time of diagnosis, presentation of the disease with neurological deficit, and type of resection. By the multivariate analysis, the extent of the disease remained as the only strong adverse risk factor for survival (HR 2.06; 95% CI = 1.38–3.07; p<0.001).

**Conclusions:**

With an organized and dedicated multidisciplinary team, the adequate outcomes can be achieved in a middle-income country setting. The presence of local residual disease after surgery and disseminated disease has a strong negative effect on survival.

## Introduction

Childhood brain tumors represent around 20% of all tumors in children and are the most common form of pediatric solid tumors [1]. The incidence rate of the primary brain and other central nervous system (CNS) tumors in children varies among regions and countries. In Europe, it has been estimated to be 2.99, and in the United States of America 6.06 per 100,000 population [2, 3]. In the high-income countries (HIC) the detailed data on pediatric patients with various tumors are derived from population cancer registries. In the low and middle-income countries (LMIC) the data about pediatric brain tumors incidence, treatment, and outcome are variable and scarce. Furthermore, the coverage of children with cancer registry is low in LMIC [4].

With the adequate treatment consisting of surgery, radiotherapy, and chemotherapy, pediatric patients with brain tumors have had improved outcomes over the last decades. Around 70% of children with CNS tumors will survive for 5 years in developed countries [5, 6]. Still, however, brain tumors remain the most common cause of death in children with cancer and further improvement of treatment modalities is necessary. Young age at diagnosis, a high histological grade along with aggressive tumor subtypes, inoperable localization, or delays in diagnosis and treatment, resulting in wider disease dissemination may contribute to a dismal outcome [7, 8]. Due to the rarity of the disease and the complexity of its management, it is recommended to treat pediatric patients with brain tumors in tertiary referral centers with a great experience in the field and coordination of a multidisciplinary team [9].

It is widely known that pediatric brain tumors have unique features compared to adult brain tumors [10]. The new molecular classification of CNS tumors [11] is a rapidly evolving field and has transformed the knowledge and approach to pediatric patients by identifying important genes and signaling pathways that serve to drive tumor proliferation [12, 13]. The advances in molecular neuro-oncology have impacted the research field by providing more accurate diagnoses and potential therapeutic targets for these diseases [14, 15]. Despite many clinical trials being still ongoing, novel targeted therapeutic agents have shown promise in being more effective and less toxic than classical treatment and will hopefully bring a much-desired improvement in treating pediatric brain tumors.

The aim of this study was to describe the characteristics of the children with brain tumors treated in a national referral oncology institution in a middle-income country and to evaluate how the pretreatment factors and treatment itself affect the overall survival of these children.

## Materials and methods

Data on pediatric patients treated at the Institute of Oncology and Radiology of Serbia (IORS), Belgrade, between January 2007 and December 2016, were analyzed. Inclusion criteria were a diagnosis of a primary brain tumor and patients age 18 or younger at the time of diagnosis. All patients were previously diagnosed by histopathological examination, except the patients with brainstem tumors, who were diagnosed by computer tomography (CT)/ magnetic resonance (MR) imaging only. All patients who underwent surgery were operated on at the Clinic of Neurosurgery, Clinical Center of Serbia, Belgrade. Histopathological examination was performed at the same institution. All patients who were included in our study underwent adjuvant therapy—radiotherapy or chemotherapy, or both, at IORS. Pediatric patients with brain tumors who were treated by surgery only at the Clinic of Neurosurgery were not included in the analysis. The Clinic of Neurosurgery, Clinical Center of Serbia, and IORS are the national referral centers for the treatment of childhood brain tumors. Any pediatric patient in Serbia suspected of having a brain tumor was admitted to these hospitals to confirm the diagnosis and to devise a treatment strategy by a common multidisciplinary team. Our childhood CNS tumors multidisciplinary team had regular weekly meetings and was in constant communication. No decision on a patient treatment was made without the knowledge and consent of all team members. We have published data on several series of our patients with various brain tumors so far [16–18]. The exclusion criteria in this study were incomplete medical records and patients with a previous history of brain irradiation (to exclude secondary neoplasms). The study was approved by the Ethical Committee of the Faculty of Medicine, the University of Belgrade, decision No. 2650/X-5. The Ethical Committee waived the requirement for the informed consent and the data were not anonymized before the collection.

Variables retrospectively collected from the patients’ medical records were: demographic characteristics (age, gender), previous history of malignant diseases and treatment, genetic syndromes, duration of symptoms before definitive diagnosis made by operation/neuroimaging (for the patients who were not operated on) in days, presentation of disease with or without epileptic seizures, signs/symptoms of an increased intracranial pressure (nausea, vomiting, headache), neurological deficit (hemiparesis, ataxia, cranial nerve palsy, dysphasia…), hormonal abnormalities (growth hormone deficiency, precocious puberty, thyroid hormone deficiency, syndrome of inappropriate antidiuretic hormone secretion…), localization of the tumor identified by neuroimaging, operative, and radio-chemotherapy data.

The patients were grouped according to the age (≤3, 4–7, 8–13, and ≥14 years), as well as according to tumor localization (supratentorial, infratentorial, or both) to determine its impact on survival. The tumors were considered supratentorial if they were affecting the cerebral hemispheres, deep cerebral structures, the third or lateral ventricles, suprasellar or pineal region, while they were considered infratentorial if they were located in the cerebellum, brainstem, or the fourth ventricle. If they were affecting *per continuitatem* at least one of the locations in the supratentorial and in the infratentorial compartment, we considered them as both, supra- and infratentorial. In patients who underwent surgery, an experienced neuro-pathologist confirmed histopathological diagnosis based on the WHO classification 2007, since the revised molecular classification appeared in 2016 [11]. The extent of surgical resection was determined based on the surgeon’s operative report and postoperative CT/MR imaging (depending on what was available at the time) as gross total resection (GTR) and non-gross total resection (NGTR), which consisted of subtotal resection, tumor reduction, or biopsy only. Based on MR images of the brain and spine and cerebrospinal fluid cytology examination, the extent of patients’ disease was classified into three categories upon admission to IORS: no evidence disease (NED) after surgery, local residual disease (LRD), or disseminated disease (DD)—micro and/or macro-dissemination. NED patients had cerebrospinal fluid cytology examination negative for malignant cells and MR of the brain and spine without residual disease or dissemination. LRD patients had a residual tumor on the brain MR without dissemination in cerebrospinal fluid or on MR of the brain and spine. If the patient had cerebrospinal fluid cytology positive for malignant cells (micro-dissemination) or metastatic deposits were observed on MR of the brain and/or spine (macro-dissemination), the patient was added to the DD group. In 2006 the three-dimensional conformal radiotherapy technique was introduced in IORS, so all the patients who underwent radiotherapy were irradiated using that technique. If chemotherapy was administered, it was given in different regimens depending on histopathological diagnosis, the age of the patient, the extent of disease, etc., according to the international protocol used at the time. After completion of the treatment, the follow-up examinations were conducted every 3 months for 24 months, every 6 months up to 5 years, followed by annual examinations. Scheduled and organized follow-up examinations allowed prompt diagnosis of the disease relapse or progression in our patients, as well as late-effects management.

Statistical analysis: Survival probability was analyzed using the Kaplan–Meier procedure. The log-rank test was used to assess differences in survival curves. The predictive value of selected variables was assessed by the univariate and multivariate Cox proportional hazard regression model. Variables significant at the 0.05 level were further analyzed in the multivariate Cox proportional hazard regression model and considered significant if p < 0.05. For the statistical analysis, the SPSS 17.0 statistical software package (SPSS Inc, Chicago, IL, U.S.A.) was used.

## Results

Out of 215 children and adolescents with brain tumors admitted at our Institute between January 2007 and December 2016, thirty-five patients were excluded from the further analysis in accordance with the above-mentioned criteria, whether because of the incomplete medical records or a previous history of brain irradiation. Three more patients refused the treatment, three were in a too bad condition to sustain the treatment, and one continued the treatment abroad. There were 173 patients identified for the analysis.

The mean patient age at the time of diagnosis was 8.96 years (range 1–18). Out of 173 patients, 20 (11.6%) patients were younger than 3 years of age, 56 (32.4%) were in the age group of 4–7 years, 64 (36.9%) were in the 8–13 age group and 33 (19.1%) were 14 years or older. The male/female ratio was 1.08. Some of the patients with inherited genetic disorders were observed in this patients’ series: three were diagnosed with neurofibromatosis type 1, one with Gilbert’s syndrome, one with Congenital adrenal hyperplasia, one patient had Congenital cataract, and one the Heterozygous deletion at chromosome 22q11. One patient was treated operatively and by chemotherapy for Burkitt’s lymphoma in clinical stage III, three years before the diagnosis of glioblastoma. The mean duration of symptoms was 133 days (range 3–2190) before a definitive diagnosis was made by operation/neuroimaging (for the patients who were not operated on). Presentation of signs/symptoms related to an increased intracranial pressure was most frequent and appeared in 128 out of 173 patients (74%), a neurological deficit in 107 patients (61.8%), seizures in 24 patients (13.9%), and hormonal abnormalities in 19 patients (11%). Tumor location was supratentorial in 71 cases (41%), infratentorial in 93 cases (53.8%) and 9 patients (5.2%) had tumor spreading through both compartments. Out of 173 patients, 153 were operated on. The extent of the surgical resection consisted of GTR in 62 (40.5%) and NGTR in 91patients (59.5%)–biopsy in 10 (6.6%), tumor reduction in 34 (22.2%) and subtotal resection in 47 (30.7%) patients. The most frequent histology was embryonal tumors in 65 (37.6%) of our patients, followed by a high-grade glioma (HGG) in 21 (12.1%) and low-grade glioma (LGG) in 21(12.1%) patients. The number of 19(11%) patients with the unknown histology was not negligible. All of these patients had tumors located in the brainstem, with a radiologic appearance of glioma. There were 13 patients (7.5%) with ependymoma in our series, 11 patients (6.4%) with germ cell tumors, 7 patients (4.1%) with craniopharyngioma, and 16 more patients (9.2%) with various histopathological diagnoses. The extent of the disease upon admission to our Institute was NED in 58 patients (33.5%), LRD in 100 patients (57.8%), and DD in 15 patients (8.7%). Radiation therapy was conducted as a part of multimodal treatment in 164 patients (94.8%). Out of 164 patients, 87 (53.1%) received the craniospinal irradiation with total dose (TD) ranging from 24–40.25Gy standard fractionation and posterior fossa boost with TD ranging from 15–30.6Gy. Standard fractionation in the pediatric population implies daily fractions in the range of 1.5–1.8 Gy, 5 days a week, depending on the age of the patient. Seventy-seven patients (46.9%) were treated locally with median TD 54 Gy standard fractionation. The majority of our patients completed the radiotherapy without major interruptions. Only in 4 patients, the treatment was discontinued due to various reasons. Chemotherapy was administered in 116 patients (67.1%), 33 patients (28.4%) received chemotherapy as neoadjuvant (before radiotherapy), 9 (7.8%) concomitant with radiotherapy, and 93 (80.2%) as adjuvant. Chemotherapy regimens varied over the years. The main clinical characteristics and treatment data can be found in Table 1 (S1 Appendix).

**Table 1:** Patients’ clinical characteristics and treatment data.

Characteristics	No.of patients (%)
***Age group (years)***	
0–3	20 (11.6)
4–7	56 (32.4)
8–13	64 (36.9)
14–18	33 (19.1)
***Gender***	
Male	90 (52.0)
Female	83 (48.0)
***Inherited genetic disorders***	
Neurofibromatosis type 1	3 (1.7)
Gilbert’s syndrome	1 (0.6)
Congenital adrenal hyperplasia	1 (0.6)
Congenital cataract	1 (0.6)
Heterozygous deletion at chromosome 22q11	1 (0.6)
***Previous history of malignant disease***	
Burkitt’s lymphoma in CS III	1 (0.6)
***Presentation***	
**↑ ICP**	128 (74.0)
Vomiting	98 (56.6)
Headache	95 (54.9)
Nausea	30 (17.3)
**Neurological deficit**	107 (61.8)
Ataxia	54 (31.2)
Strabismus	38 (22.0)
Hemiparesis	37 (21.4)
Impaired vision	22 (12.7)
Vertigo	13 (7.5)
Facial nerve palsy	9 (5.2)
Nystagmus	8 (4.6)
Dysphasia	5 (2.9)
Dysphagia	4 (2.3)
Bladder and bowel incontinence	2 (1.2)
Hearing impairment	1 (0.6)
**Epileptic seizures**	24 (13.9)
Generalized	18 (10.4)
Partial	6 (9.2)
**Hormonal abnormalities**	19 (11.0)
SIADH	13 (7.5)
Thyroid hormone deficiency	7 (4.0)
Adrenal deficiency	4 (2.3)
Growth hormone deficiency	3 (1.7)
Precocious puberty	3 (1.7)
***Tumor location***	
Supratentorial	71 (41.0)
Infratentorial	93 (53.8)
Both	9 (5.2)
***Histopathological type (WHO 2000)***	
**Embryonal tumors**	65 (37.6)
Medulloblastoma WHO gr. IV	55 (31.8)
PNET WHO gr. IV	9 (5.2)
ATRT WHO gr. IV	1 (0.6)
**LGG**	21 (12.1)
Pilocytic astrocytoma WHO gr. I	7 (4.0)
Diffuse astrocytoma WHO gr. II	7 (4.0)
Pleomorphic xanthoastrocytoma WHO gr. II	5 (2.9)
Oligodendroglioma WHO gr. II	2 (1.2)
**HGG**	21 (12.1)
Anaplastic astrocytoma WHO gr. III	4 (2.3)
Glioblastoma WHO gr. IV	17 (9.8)
**Ependymoma**	13 (7.5)
WHO gr. II	7 (4)
WHO gr. III	6 (3.5)
**Germ cell tumors**	11 (6.4)
Germinoma	9 (5.2)
NGGCT	2 (1.2)
**Craniopharyngioma**	7 (4.1)
**Other**	16 (9.2)
**Unknown**	19 (11.0)
***Surgery***	153 (88.4)
**GTR**	62 (40.5)
**NGTR**	91 (59.5)
B	10 (6.6)
TR	34 (22.2)
STR	47 (30.7)
***Extent of disease***	
NED	58 (33.5)
LRD	100 (57.8)
DD	15 (8.7)
***Radiotherapy***	164 (94.8)
CSI + local	87 (53.1)
Local	77 (46.9)
***Chemotherapy***	116 (67.1)
Neoadjuvant	33 (28.4)
Concomitant	9 (7.8)
Adjuvant	93 (80.2)

CS–clinical-stage, SIADH—**s**yndrome of inappropriate antidiuretic hormone secretion, ICP–intracranial pressure, WHO–World Health Organization, gr.–grade, PNET–Primitive neuro-ectodermal tumor, ATRT–Atypical teratoid rhabdoid tumor, LGG–Low-grade glioma, HGG–High-grade glioma, NGGCT—Non-germinomatous germ cell tumor, GTR–gross total resection, NGTR–non-gross total resection, STR–subtotal resection, TR–tumor reduction, B–biopsy, NED–no evidence disease, LRD–local residual disease, DD–disseminated disease, CSI–craniospinal irradiation.

**Fig 1:**
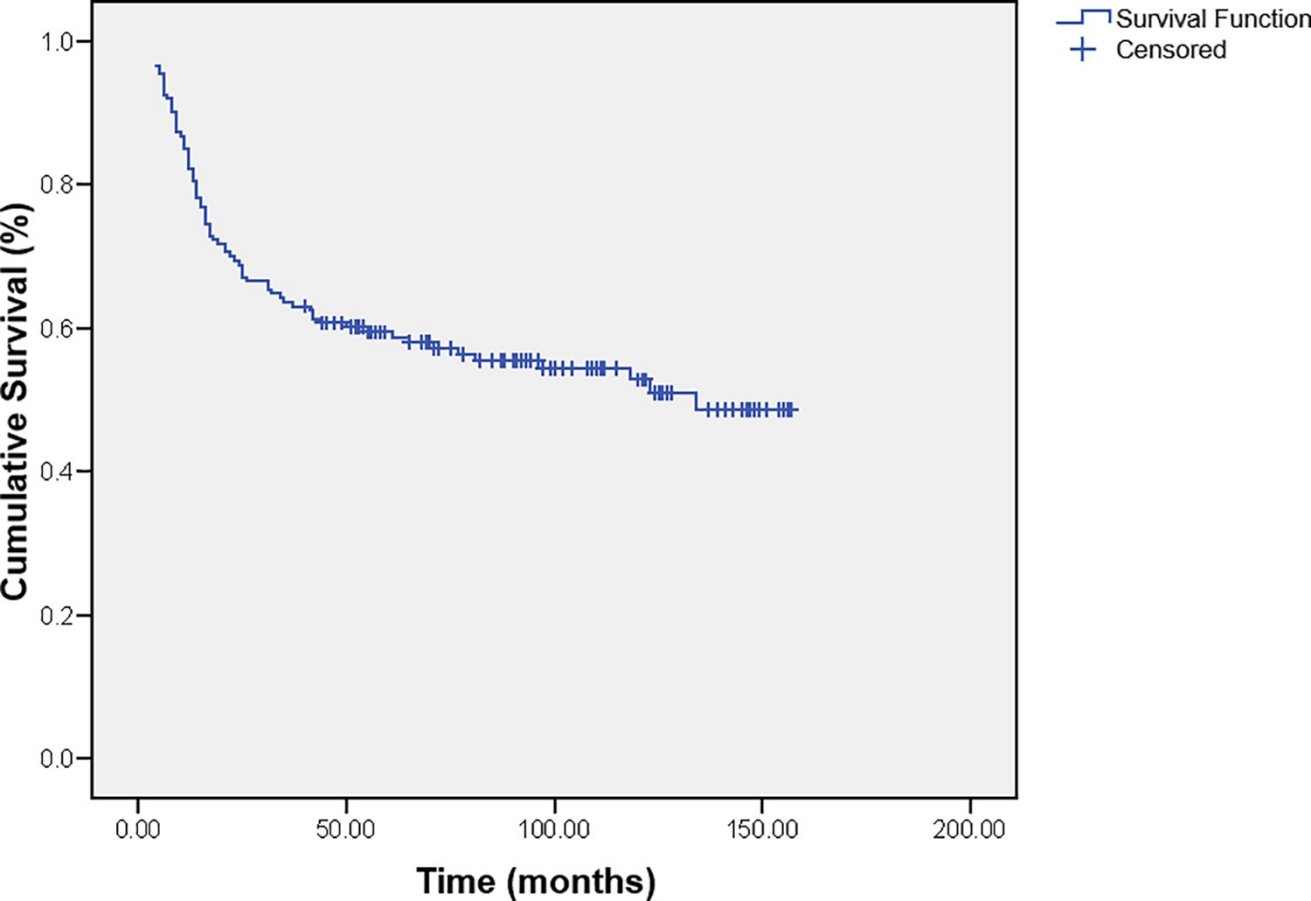
Cumulative survival of children with brain tumors.

The mean survival time of all the children was 94.5months (95% Confidence Interval (CI) 84.2–104.7). The 2-, 5- and 10-year overall survival (OS) probabilities were 68.8% ± 3.5, 59.4%± 3.7 and 52.8% ± 4.2 (Fig 1).

**Fig 2:**
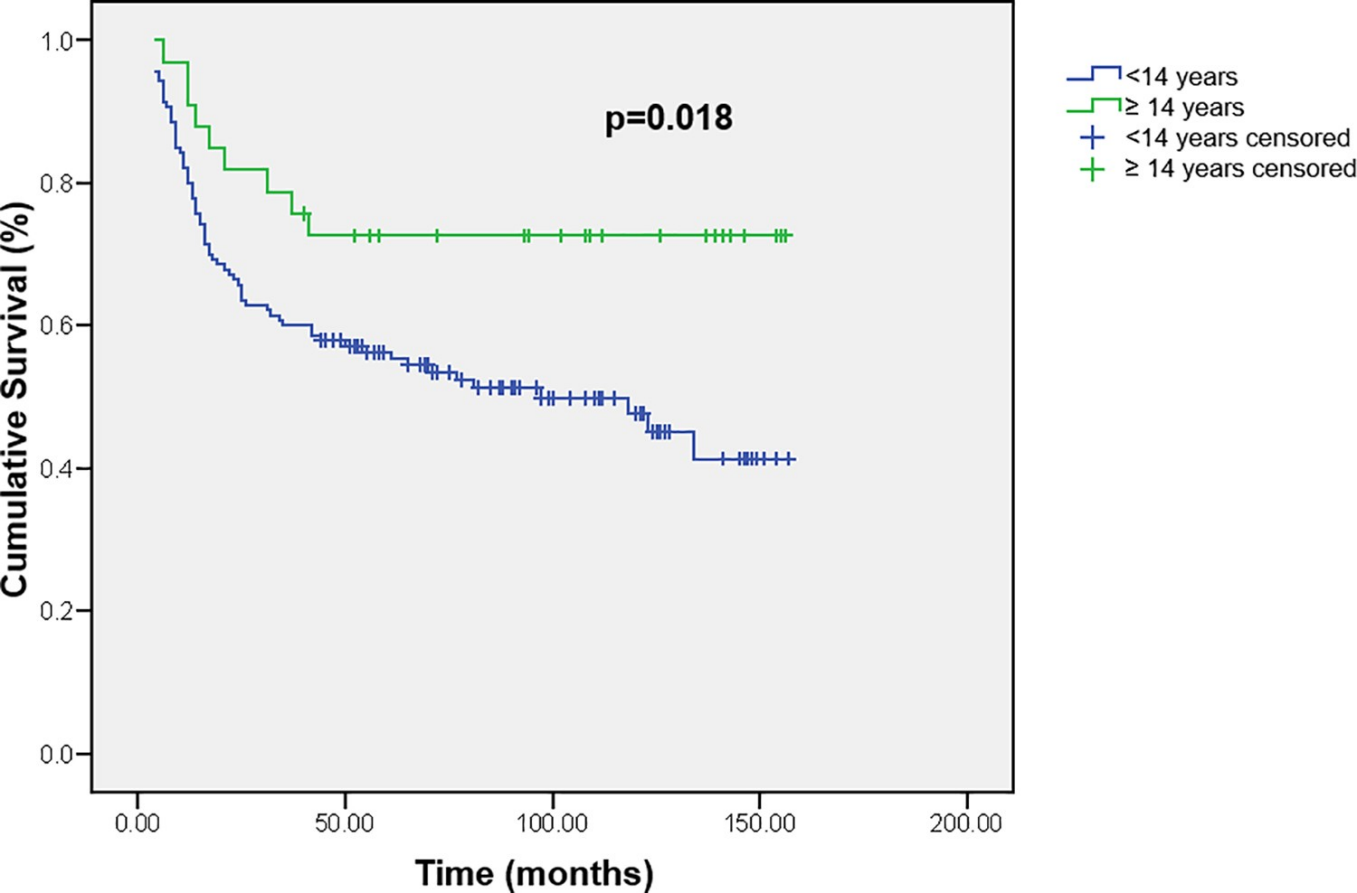
Cumulative survival of children with brain tumors according to age.

There were no statistically significant differences in the OS based on patients’ gender or age, except that patients who were 14 years or older at the time of diagnosis lived longer compared to all other younger children (p = 0.018, Fig 2).

**Fig 3:**
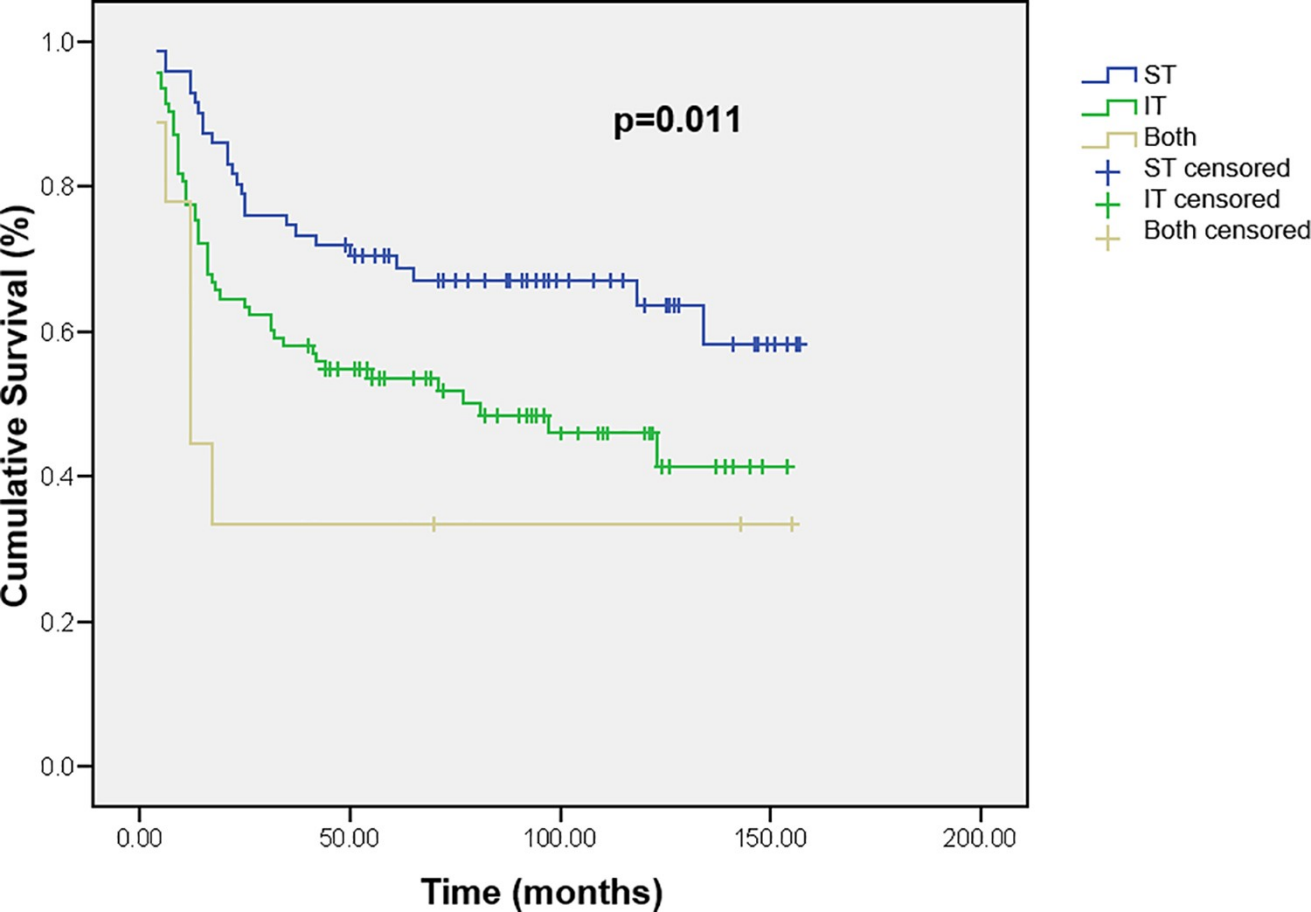
Cumulative survival of children with brain tumors according to tumor location. ST—supratentorial tumors, IT—infratentorial tumors, Both—tumors with both (supratentorial and infratentorial) locations involvement.

Tumor location was also a parameter with a significant impact on survival. Patients with supratentorial tumors had a mean survival time of 111.0 ± 7.5 months, compared with 83.3± 7.1 months for the patients with infratentorial tumors and 58.7± 22.7 months for the tumors in both compartments (p = 0.011) (Fig 3).

**Fig 4:**
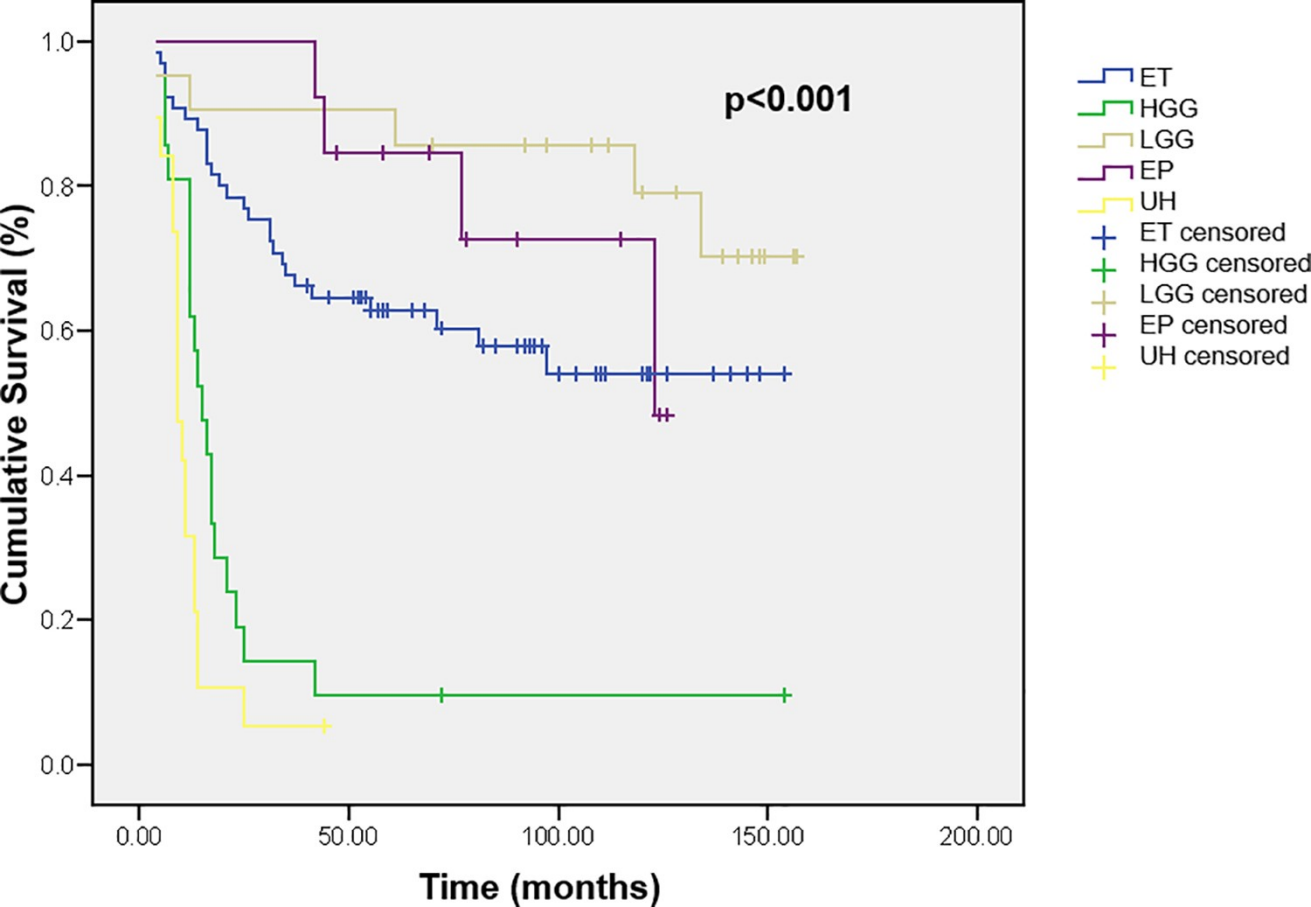
Cumulative survival of children with brain tumors according to tumor histopathological type. ET–embryonal tumors, HGG–high-grade glioma, LGG–low-grade glioma, EP–ependymoma, UH–unknown histopathological type.

The histopathological type of tumor was also a significant predictor of survival in our series. Patients with the unknown histopathology (brainstem glioma) and HGG had a shorter life than the other most frequent tumor types–embryonal tumors, ependymoma, and LGG (p<0.001, Fig 4). 1, 2-, and 5-year survival probabilities of children with the unknown histopathology were 31.6% ± 10.7, 10.5% ± 7.0, and 0.0%, and children with HGG 61.9% ± 10.6, 19.0% ± 8.6, and 9.5% ± 6.4. Children with the embryonal tumors had a 5-year survival probability of 62.7% ± 6.0, children with ependymoma 84.6% ± 10, and with LGG 90.5% ± 6.4.

**Fig 5:**
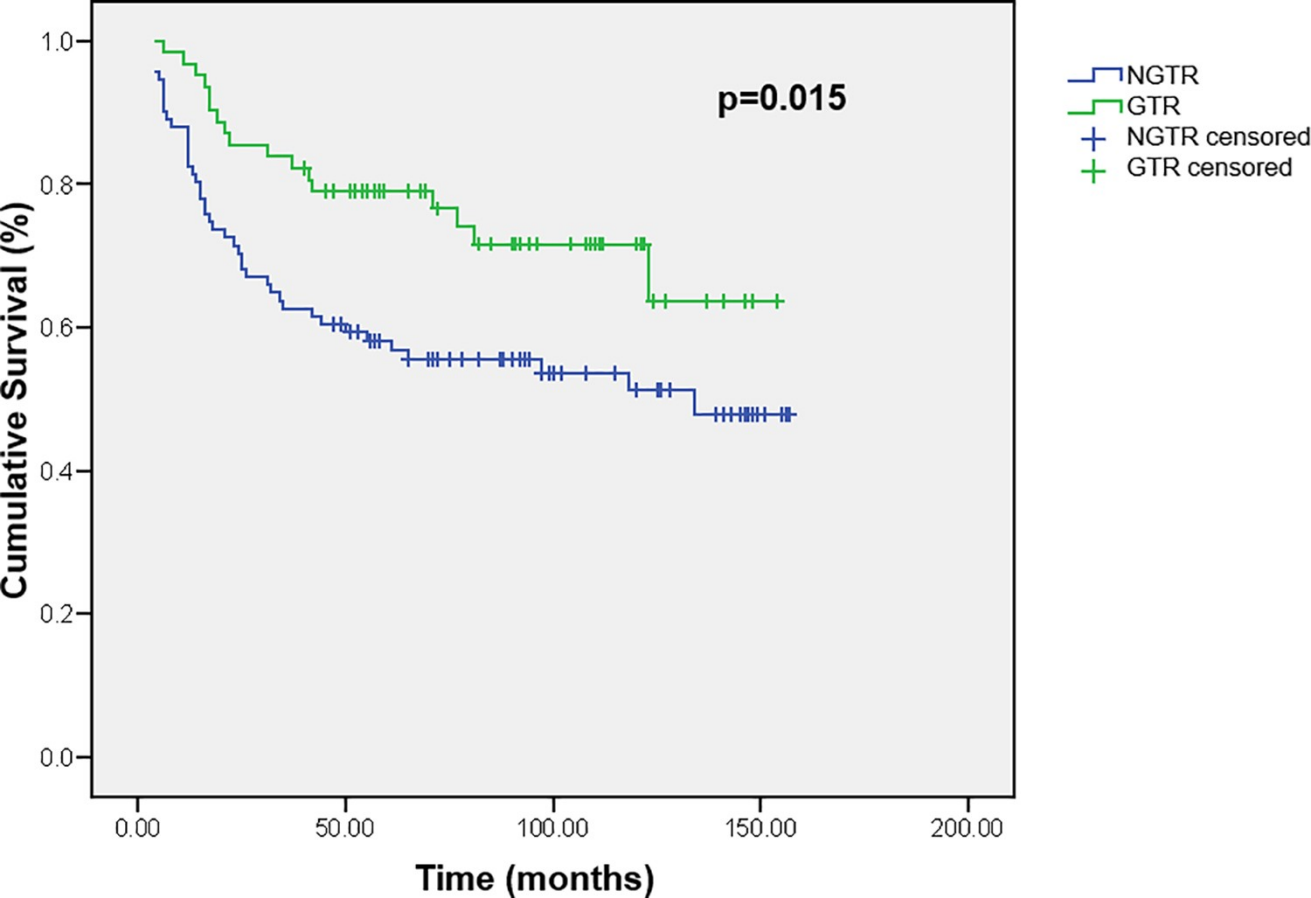
Cumulative survival of children with brain tumors according to type of resection. GTR–gross total resection, NGTR–non-gross total resection.

The factor found to be associated with the improved outcome was the extent of resection. Survival of the children who underwent GTR was significantly longer than the children in whom lesser degrees of resection were achieved (p = 0.015). Five- and ten-year survival of children with GTR was 79.0% ±5.2 and 71.6% ± 6.2, compared to 58.1% ± 5.2 and 51.2% ± 5.7 in children without GTR (Fig 5).

**Fig 6:**
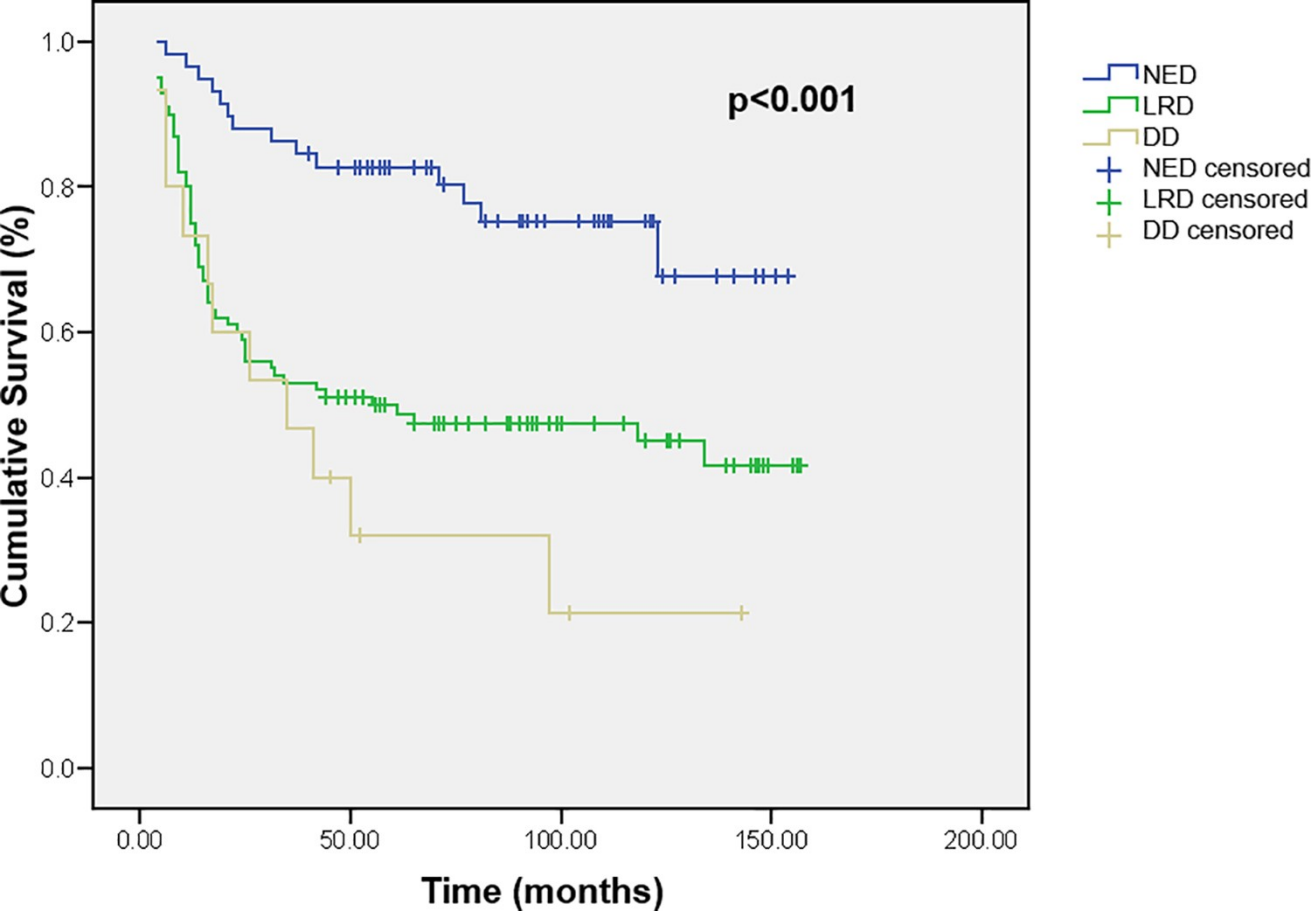
Cumulative survival of children with brain tumors according to extent of the disease. NED–no evidence disease, LRD–local residual disease, DD–disseminated disease.

The extent of the disease is also a very important parameter found to be associated with the survival. Patients with NED upon admission to IORS had a mean survival of 123.1± 7.2 months, compared with 82.1± 7.0 months in patients with LRD and 55.6± 14.0 months in patients with DD (p<0.001) (Fig 6).

We did not analyze the impact of radiotherapy and chemotherapy on the survival as these modalities vary according to the histological diagnosis and protocol.

By the univariate analysis, factors predicting poor outcome in our series were the presentation of the disease with hormonal abnormalities, tumor location, and the extent of disease, while the factors predicting a better outcome were age at the time of diagnosis, presentation of the disease with neurological deficit, and type of resection. Other factors such as gender, presence of inherited genetic disorders, duration of symptoms before definitive diagnosis, presentation of disease with an increased intracranial pressure, or epileptic seizures had no prognostic impact on the survival (Table 2).

**Table 2:** Predictive factors of OS in 173 pediatric patients with brain tumors–Cox proportional hazard regression models.

	Univariate analysis			Multivariate analysis		
Variable	HR	95% CI	p	HR	95% CI	p
**Age at the time of diagnosis**	0.95	0.90–0.99	0.044			
**Gender**	0.84	0.54–1.32	0.453			
**Inherited genetic disorders**	0.85	0.31–2.32	0.745			
**Duration of symptoms before definitive diagnosis**	0.98	0.90–1.00	0.063			
**Presentation of the disease with increased intracranial pressure**	1.23	0.75–2.02	0.410			
**Presentation of the disease with epileptic seizures**	0.84	0.46–1.56	0.585			
**Presentation of the disease with neurological deficit**	0.62	0.38–1.00	0.005			
**Presentation of the disease with hormonal abnormalities**	4.05	1.28–2.86	0.002			
**Tumor location**	1.82	1.25–2.66	0.002			
**Type of resection**	0.52	0.29–0.91	0.022			
**The extent of the disease**	2.14	1.50–3.06	<0.001	2.06	1.38–3.07	<0.001

HR–Hazard Ratio, CI–Confidence Interval.

By the multivariate analysis, the extent of the disease remained the only strong adverse risk factor for OS (HR 2.06; 95% CI = 1.38–3.07; p<0.001).

## Discussion

We have presented a national referral oncology institution experience of all pediatric brain tumors treated over a 10-year period in Serbia.

In order to compare our results, using the literature search, we have identified around 40 worldwide pediatric series of CNS tumors published in the last 20 years, including 8 European. Few studies report clinical profile, treatment modalities, and/or survival data (S1 Table), as well as our study.

Our series showed a slight predominance of males (male to female ratio 1.08:1) which is in agreement with the data from the most of the published series and the data from the United States population registry [1]. In a series from Greece [19] the male to female ratio was 1.15:1, from Denmark [20] 1.12:1, and Poland [21] 1.24:1.

The mean patient age at the time of diagnosis was 8.96 years and the most affected age group was 8–13 years. Similar numbers have been reported by some series from Europe [20–22] and large series from Africa and Asia [23–25].

Some of the patients with inherited genetic disorders were observed in our group, as well as one patient with a history of malignant disease. The most frequent genetic disorder was neurofibromatosis type 1 in three of our patients. The appearance of the neurocutaneous syndrome was identified in a series of patients with CNS tumors from New Zealand [26], Brazil [27], and Portugal [22], with neurofibromatosis type 1 as being the most common. The Portuguese study also reported a previous history of malignant diseases in two of their patients treated by the cranial irradiation before a diagnosis of brain tumor, which can indicate the secondary nature of the disease. Our patient was diagnosed with Burkitt’s lymphoma three years before the diagnosis of glioblastoma. The patient was previously treated operatively and by chemotherapy, without irradiation. To our knowledge, no similar case has been reported in the literature.

The mean duration of symptoms was 133 days (around 4.5 months) before a definitive diagnosis in our group of patients. The mean interval from the symptom onset to diagnosis was 2 months in a study from New Zealand [26], 6 months in a Sudanese [28], and 6.3 months in a Portuguese study [22]. Other series from developing countries reported even longer intervals, like in Nigeria with a mean interval to presentation of 21.5 months for children without hydrocephalus, or in Uganda where time to diagnosis is very prolonged due to the lack of neurosurgical staff [29–31]. Despite the modern imaging methods, a long symptom-to-diagnosis interval can be observed in pediatric patients with primary brain tumors in developed countries. Fukuoka et al. [32] found high tumor grade to be the only significant factor for a short prediagnostic symptomatic interval in Japanese children. Shortening the timeframe to diagnosis and treatment of pediatric brain tumors remains a challenge worldwide.

A French study from Bauchet et al. [33] reported signs and symptoms of intracranial hypertension to be the most frequent presentation of the disease among children younger than 15 years, followed by neurological deficit and seizures, just like in our series. In the study from New Zealand, Monteith et al. [26] reported a symptomatic hydrocephalus as the most common finding, as well as the studies from Sudan, Uganda, India, and Nepal [28, 29, 34, 35].

The most frequent tumor location in our patients was the infratentorial compartment, just like in some series from and outside Europe [31, 33, 36–38]. However, the series from Greece, Portugal, Australia, New Zealand, and Japan demonstrated the supratentorial compartment as a predominant site [19, 22, 26, 32, 39].

The most common brain tumors among our patients were embryonal tumors (37.6%), followed by equal numbers of HGG (12.1%) and LGG (12.1%). Medulloblastoma was the most common individual histology. Our series does not reflect previously published European data [2] and the majority of published series which reported glial tumors as the most frequent tumor type, followed by embryonal tumors and ependymoma [19, 21, 24, 28, 33, 40, 41]. This is due to the fact that our series consisted only of patients who received some form of adjuvant treatment after surgery in our institution. In that way, not a negligible number of cases, especially with LGG that required operation only, were not included in our analysis.

In our series, the extent of surgical resection consisted of GTR in 40.5% and NGTR in 59.5% of the patients. The extent of resection was determined based not only on the surgeon’s operative report but also on the postoperative imaging. In the Portuguese study [22] GTR was achieved in 63% of their patients, without further explaining how the degree of resection was determined. Also, in the series from Nigeria with GTR in 62.5% of the patients [31]. Authors from Nepal reported GTR in 62.9% without routinely performed postoperative imaging [35]. Authors from New Zealand reported a lower rate of GTR in 35.5% of their patients, similar to our results, determined by an immediate (within 72 hours) postoperative imaging in the majority [26]. In Uganda, 1.2% of tumors were biopsied and only 25.5% more resected [36].

Monteith et al. [26] reported 10.2% of patients with disseminated disease at the presentation. The majority of these cases were spinal metastases from medulloblastoma. Upon the admission to IORS, there were 8.7% of patients with DD, despite a relatively large proportion of medulloblastoma in our series.

All published studies reported that the survival of the children with brain tumors has improved in recent years. In our study, the mean survival time was 94.5months (7.9 years), and the 2-, 5- and 10-year OS probabilities were 68.8%, 59.4%, and 52.8%, respectfully. Our survival rates are comparable with the results from other centers and countries. In a study from Poland, Pogorzala et al. [21] reported similar findings with 5- and 10- survival probabilities of 60.9% and 58.2%. Researchers from Australia and Sweden reported higher 5-year survival rates of 80% and 76% [39, 41], and Nigeria, Tunisia, and Sudan reported lower rates of 47%, 45%, and 13%, respectfully [24, 27, 30]. We have to emphasize the fact that our series consisted only of patients who received some form of adjuvant treatment after the surgery. In that way, not a negligible number of cases with a good prognosis that required operation only were not included in our analysis. We are certain that by the inclusion of those patients our results would have been even better.

We did not find significant differences in OS based on patients’ gender or age, except the fact that patients who were 14 years or older at the time of diagnosis lived longer compared to others, and the age was found to be a factor predicting a better outcome in an univariate analysis. In a study by Lannering et al. [41] infants (<1 year of age) had inferior survival compared to older children, and especially children over 10 years at the diagnosis.

Presentation of the disease with hormonal abnormalities was found to be a factor predicting poor outcome in our series, while the presentation with neurological deficit predicted a better outcome in an univariate analysis. The reason for this may be that children with evident neurological signs get diagnostic imaging and subsequent treatment faster than children with other symptoms and especially with hormonal abnormalities, which can be caused by many other conditions leading to misdiagnosis.

Location of brain tumor was found to be a factor predicting a poor outcome in an univariate analysis of our study. Our patients with supratentorial tumors lived longer than patients with tumors in infratentorial location and patients with tumors in both compartments, as well as in literature [24].

In our study, the patients with the unknown histopathology (brainstem glioma) and HGG had a shorter life than embryonal tumors, ependymoma, and LGG. The Poland’s study [24] also reported survival rates according to histology in their patients and our results were quite similar. We have found higher survival rates for patients with ependymoma, just like the authors from Australia [39]. However, they have achieved better results with HGG. They believe they owe their good results to the aggressive nature of the surgery and radiotherapy, as well as routinely offered “second look” surgeries of residual tumor, which is not a rare practice in our circumstances, too.

In our series, the survival of children who underwent GTR was longer than the children in whom lesser degrees of resection were achieved. The extent of resection was also a factor found to be associated with the improved outcomes in an univariate analysis. The extensive resection is a treatment goal for most children with brain tumors, however, the complete resection is not usually feasible for deep-seated, infiltrative lesions [42].

Our patients with NED after surgery and upon the admission to IORS lived longer than patients with LRD and DD. The high statistical significance of the extent of disease on survival has been proven in an univariate and multivariant analysis. These findings are in concordance with many reports of pediatric brain tumors from the literature and the importance of the complete resection/absence of residual tumor remains most prominent in glioma and ependymoma management [43, 44]. It is also widely known that children with disseminated tumors have less favorable outcomes, although in medulloblastoma some progress has been made [45].

Limitations of this study were the inclusion of children with brain tumors treated with some form of adjuvant treatment after surgery and not all children with brain tumors in Serbia, which makes this study a retrospective series of pediatric patients treated at a single institution, although the national referral cancer center, IORS. Unfortunately, not a negligible number of cases with a good prognosis that required operation only were not included in our analysis, which would make our outcomes even better. With its population of 6.945 million, the Republic of Serbia is listed as a middle-income country on the World bank list [46] and the precise data reporting of children with CNS tumors has not been established yet, as in some other countries of the Southern and Eastern Europe [47]. However, the program adopted by the Government for improving cancer control in Serbia for the period 2020–2022 envisages the establishment of a special registry for cancer in children, with whom we will hopefully overcome these flaws and be able to compare our results more precisely.

Though the present study was a hospital-based analysis, the results are similar to the data from other series and the well-established population-based study reports. Our treatment outcomes are not much inferior to the data from developed countries and are superior to the data from some developing countries. With the future implementation of a national pediatric cancer registry, we are expecting better reporting and even the survival of children with brain tumors [48]. Having in mind the importance of the extent of disease on survival, there is hope that with further improvement of the amount of resection, together with the improvement of other treatment modalities, even better outcomes are possible. Also, by implementing new classification with molecular characteristics of pediatric brain tumors and specific target therapeutical agents in the future, we are expecting breakthrough improvements in the prognosis of these patients, just like in some other diseases [49, 50]. Further collaborative research worldwide is needed to obtain better knowledge and understanding of pediatric brain tumors.

## Conclusion

Children with brain tumors have a chance for long-term survival. With an organized and dedicated multidisciplinary team, the adequate outcomes can be achieved in a middle-income country setting. The presence of local residual disease after surgery and disseminated disease has a strong negative effect on survival.

## Supporting information

S1 Appendix Minimal dataset. (XLSX)

S1 Table Published series of pediatric brain tumors worldwide in the last 20 years that reported clinical profile, treatment modalities, and/or survival data. CNS–Central nervous system, WHO–World Health Organization.
(DOCX)
