# Supplementary material for: Clinical profile, treatment and outcome of pediatric brain tumors in Serbia in a 10-year period: A national referral institution experience
Source: PLoS One. 2021 Oct 26;16(10):e0259095. doi: 10.1371/journal.pone.0259095 (PMC8547703; doi:10.1371/journal.pone.0259095)
Supplement: S1 Table — CNS–Central nervous system, WHO–World Health Organization. (DOCX) [file pone.0259095.s002.docx]

|  | Author/year | Journal | Country | No. of institutions | Time (years) | No. of patients | Patients’ Age (years) | Type of Tumor | Histopathological Classification | Data |
| --- | --- | --- | --- | --- | --- | --- | --- | --- | --- | --- |
| 1 | Pongtanakul B et al, 2020 [48] | Journal of Neuro-Oncology | Thailand | Multi | 3  1 | 300  168 | ≤15 | Primary CNS tumors | WHO 2000 | Demography, pathology, survival |
| 2 | Elhassan MA et al., 2019 [28] | Child’s Nervous System | Sudan | 1 | 16 | 62 | <15 | Primary CNS tumors | WHO 2007 | Demography, pathology, treatment, survival |
| 3 | Madhavan R et al., 2018 [34] | Indian Journal of Cancer | India | 1 | 5 | 250 | <18 | Primary brain tumors | WHO 2007 | Demography, pathology, treatment |
| 4 | Santos MM et al., 2016 [22] | Child’s Nervous System | Portugal | 1 | 11 | 215 | ≤18 | Primary CNS tumors | WHO 2007 | Demography, pathology, treatment |
| 5 | Azad TD et al., 2015 [35] | World Neurosurgery | Nepal | 1 | 5 | 39 | <18 | Primary CNS tumors | WHO 2007 | Demography, pathology, treatment |
| 6 | Hatef J et al., 2014 [36] | World Neurosurgery | Uganda | 1 | 4 | 129 | ≤19 | Primary CNS tumors | WHO 2007 | Demography, pathology, treatment |
| 7 | Stagno V et al., 2014 [29] | Child’s Nervous System | Uganda | 1 | 10 | 172 | ≤18 | Primary brain tumors | WHO 2007 | Demography, clinical, pathology, treatment, survival |
| 8 | Pogorzala M et al., 2014 [21] | Anticancer Research | Poland | 1 | 11 | 110 | ≤18 | Primary brain tumors | WHO 2007 | Demography, pathology, treatment, survival |
| 9 | Fukuoka K et al., 2014 [32] | Pediatrics International | Japan | Multi | 19 | 127 | <15 | Primary CNS tumors | WHO 2007 | Demography, pathology, survival |
| 10 | Uche EO et al., 2013 [31] | Child’s Nervous System | Nigeria | 1 | 13 | 40 | ≤16 | Primary brain tumors | ? | Demography, clinical, pathology, treatment, survival |
| 11 | Ramanan M et al., 2012 [39] | Journal of Clinical Neuroscience | Australia | 1 | 12 | 313 | ≤17 | Primary brain tumors | WHO 2007 | Demography, pathology, survival |
| 12 | Pinho RS et al., 2011 [27] | Journal of Pediatric Hematology and Oncology | Brazil | 1 | 21 | 741 | <21 | Primary CNS tumors | WHO 2007 | Demography, clinical, pathology |
| 13 | El-Gaidi MA, 2011 [30] | Pediatric Neurosurgery | Egypt | Multi | 4 | 451 | ≤14 | Primary brain tumors | WHO 2007 | Demography, clinical, pathology |
| 14 | Nikitovic M et al., 2011 [16] | Journal of BUON | Serbia | 1 | 10 | 212 | ≤18 | Primary brain tumors | WHO 2007 | Demography, pathology, treatment, survival |
| 15 | Lannering B et al., 2009 [41] | Acta Paediatrica | Sweden | Multi | 20 | 1479 | ≤14 | Primary brain tumors | WHO 2007 | Demography, pathology, survival |
| 16 | Bauchet L et al., 2009 [33] | Journal of Neuro-Oncology | France | Multi | 3 | 1017 | ≤19 | Primary CNS tumors | WHO 2000 | Demography, clinical, pathology, treatment |
| 17 | Bellil S et al., 2008 [24] | Pediatric Neurosurgery | Tunisia | 1 | 15 | 492 | ≤15 | CNS tumors | WHO 2007 | Demography, pathology, survival |
| 18 | Monteith SJ et al., 2006 [26] | Journal of Clinical Neuroscience | New Zealand | Multi | 10 | 166 | ≤14 | CNS tumors | ? | Demography, clinical, pathology, treatment, survival |
